# Hypoadiponectinemia and the presence of metabolic syndrome in patients with chronic kidney disease: results from the KNOW-CKD study

**DOI:** 10.1186/s13098-016-0191-z

**Published:** 2016-11-14

**Authors:** Chang-Yun Yoon, Yung Ly Kim, Seung Hyeok Han, Tae-Hyun Yoo, Su-Ah Sung, Woo-kyung Chung, Dong-Wan Chae, Yong-Soo Kim, Curie Ahn, Kyu Hun Choi

**Affiliations:** 1 Department of Internal Medicine, College of Medicine, Institute of Kidney Disease Research, Yonsei University, 50 Yonsei-ro, Seodaemun-gu, Seoul, 120-752 Republic of Korea; 2 Department of Internal Medicine, Eulji General Hospital, Seoul, Republic of Korea; 3 Department of Internal Medicine, Gachon University School of Medicine, Incheon, Republic of Korea; 4 Department of Internal Medicine, Seoul National University Bundang Hospital, Gyeonggi-do, Seongnam, Republic of Korea; 5 Department of Internal Medicine, Seoul St. Mary Hospital, Catholic University of Korea, Seoul, Republic of Korea; 6 Department of Internal Medicine, Seoul National University Hospital, Seoul, Republic of Korea

**Keywords:** Adiponectin, Chronic kidney disease, Metabolic syndrome

## Abstract

**Background:**

In patients with chronic kidney disease, metabolic syndrome has been demonstrated to be the culprit behind diverse complications. Adiponectin is known to have anti-atherogenic and cardio-protective effects. Meanwhile, the relationship between adiponectin and metabolic syndrome in patients with chronic kidney disease has not been clarified. The aim of this study was to elucidate the relationship between adiponectin level and metabolic syndrome in patients with chronic kidney disease.

**Methods:**

The KoreaN Cohort Study for Outcome in Patients with Chronic Kidney Disease is a cohort study that enrolled subjects with chronic kidney disease throughout South Korea. From February 2011 to July 2014, data were collected from 1332 patients with chronic kidney disease.

**Results:**

The mean age of the patients was 53.5 years and 803 patients (60.7%) were men. The median adiponectin level was 10.7 μg/mL and 585 (44.3%) patients had metabolic syndrome. In multiple linear regression analysis, log adiponectin was positively associated with high-density lipoprotein cholesterol levels (β = 0.006), whereas it was negatively associated with serum albumin (β = −0.284), triglyceride (log β = −0.288), high sensitivity C-reactive protein (log β = −0.058) levels and estimated glomerular filtration rate (β = −0.005). Multiple logistic regression analysis indicated that low adiponectin level was independently associated with a higher risk of metabolic syndrome (per 1 μg/mL increase; odds ratio = 0.953, 95% confidence interval = 0.898–0.970, *P* < 0.001) after adjustment for multiple confounding factors.

**Conclusions:**

Hypoadiponectinemia is independently associated with the presence of metabolic syndrome in patients with chronic kidney disease.

## Background

Metabolic syndrome is defined as the aggregation of non-traditional risk factors, including central obesity, hypertension, hyperglycemia, hypertriglyceridemia, and decreased high-density lipoprotein cholesterol (HDL-C) [1]. The clinical significance of metabolic syndrome is its association with endocrinologic derangement, cardiovascular events [2], and renal impairment [3] in the general population. In addition, unfavorable clinical relationships were also reported in patients with chronic kidney disease on maintenance dialysis [4].

Adiponectin, which is released from adipocytes [5], is known to have anti-atherogenic and cardio-protective effects. It is chiefly related to insulin resistance and systemic inflammation, which are important factors for non-traditional risks of adverse outcome in chronic kidney disease [6]. Hypoadiponectinemia is related to metabolic syndrome [7] and increased intima-media thickness, which is a well-known early indicator of atherosclerosis in the general population [8] and in patients with chronic kidney disease [9]. However, the relationship between adiponectin level and metabolic syndrome in patients with chronic kidney disease has not been definitely clarified [10]. Decreased estimated glomerular filtration rate (eGFR), which is a well-known risk factor of metabolic syndrome, is significantly and independently related to hyperadiponectinemia in patients with chronic kidney disease [11]. In addition, several medications affecting adiponectin level and metabolic derangements such as statins or fibrates were not considered in most previous research studies. Regarding the important impact on clinical outcomes of metabolic syndrome in patients with chronic kidney disease, clarification of the relationship between metabolic syndrome and adiponectin level is mandatory.

Therefore, the aim of this study is to clarify the determinant factors of adiponectin level, and elucidate the relationship between adiponectin level and metabolic syndrome in patients with chronic kidney disease.

## Methods

### Subjects

The study has a cross-sectional design, using the database system of the KoreaN Cohort Study for Outcome in Patients with Chronic Kidney Disease (KNOW-CKD), a nationwide multicenter prospective cohort including chronic kidney disease stage 1–5 pre-dialysis patients from February 2011 to July 2014. KNOW-CKD was implemented to investigate the pathophysiologic mechanisms of renal or cardiovascular outcomes in Korean chronic kidney disease populations. The detailed design and methods of the KNOW-CKD were previously published [12]. Among 1528 patients, 73 patients were excluded due to unavailable eGFR or adiponectin data, and 133 patients were also excluded due to missing data on blood pressure, waist measurement, triglycerides, HDL-C, or glucose. A total of 1322 patients were included in the final analysis.

### Data collection

All data analyzed in this study were collected at the time of enrollment. The demographic data were collected by experienced doctors and nurses from the study subjects. Body mass index was calculated as weight in kilograms divided by the square of height in meters [13], and the measurements of baseline blood pressure were performed in the clinic using an electronic sphygmomanometer. The use of medication at the time of enrollment was determined by a specialized nurse for KNOW-CKD in accordance with the study protocol.

A total 10 mL of whole blood was collected using a serum separation tube (SST) and centrifuged within 1 h after collection for the separation of serum prior to delivery to the central laboratory (Lab Genomics, Korea) for measuring creatinine. Laboratory data included complete blood cell counts, glucose, uric acid, blood urea nitrogen, serum creatinine, calcium and phosphate, lipid profiles, serum albumin, and high sensitivity C-reactive protein (hs-CRP) level at the central laboratory. Serum creatinine was measured by an IDMS-traceable method. Calcium levels were corrected by using the serum albumin level. Another 15 mL of the first-voided urine was also collected to analyze urinary levels of albumin and creatinine at the central laboratory. Albuminuria was determined by urinary albumin-to-creatinine ratio (UACR) from spot urine samples, and eGFR was calculated using the four-variable modification of diet in renal disease (MDRD) equation including age, sex, race, and serum creatinine level [14]. Plasma adiponectin (Adipogen AG, Liestal, Switzerland) was measured using enzyme-linked immunosorbent assays. The Adiponectin (human) ELISA Kit, which was used in analysis of KNOW-CKD cohort subjects, is a total adiponectin kit that detects the total level of low-, middle-, and high-molecular-weight adiponectin. Other methods were described in our previous study [12].

### Diagnosis of metabolic syndrome

The harmonizing definition [1] bases the diagnosis of metabolic syndrome on the presence of three or more of the following: (1) waist circumference (abdominal obesity) >89 cm in men or >80 cm in women, (2) triglyceride ≥150 mg/dL or taking medication for dyslipidemia, (3) HDL-C <40 mg/dL in men or <50 mg/dL in women or taking medication, (4) systolic blood pressure ≥130 mmHg and/or diastolic blood pressure ≥85 mmHg or taking medication for hypertension, (5) fasting glucose ≥100 mg/dL or taking medication for diabetes. Defining thresholds for abdominal obesity are based on the Asian population [15].

### Statistical analyses

Continuous variables were represented by using mean ± standard deviation or the median (interquartile range), while categorical variables were represented as the number of subjects (percentage). Normality of distribution was ascertained by the Kolmogorov–Smirnov test. To compare differences between patients with and without metabolic syndrome, Student’s *t* test or the Mann–Whitney *U*-test was used for continuous variables and the Chi-square test for categorical variables. Univariate and multiple linear regression analyses were performed to determine independent factors associated with adiponectin level. Regarding the distribution of variables, adiponectin level, triglyceride, hs-CRP, and UACR were transformed to natural logarithm values for linear regression analysis. The independent predictive role of adiponectin level for the presence of metabolic syndrome was confirmed by multiple logistic regression analysis, which included significant variables in the comparison of baseline characteristics according to the presence of metabolic syndrome and multiple linear regression analysis. For further investigation beyond multiple logistic regression analysis, a cubic spline model was developed to illustrate systemic relations between adiponectin level and the risk of metabolic syndrome. We also conducted baseline subgroup analyses stratified by cause of chronic kidney disease, sex, age, diabetes mellitus, body mass index, UACR, eGFR, statin use, and angiotensin II receptor blocker (ARB). The trend for the mean adiponectin level across the numbers of metabolic syndrome components was verified by the trend test. Statistical analysis was performed with IBM SPSS Statistics for Windows version 23.0 (IBM SPSS Inc., Chicago, IL, USA). *P* values less than 0.05 were considered significant.

## Results

### Baseline characteristics according to the presence of metabolic syndrome

The baseline characteristics are shown in Table 1. The mean age of the study population was 53.5 ± 12.4 years and 803 (60.7%) patients were men. There were 153 (11.6%), 243 (18.4%), 511 (38.7%), 312 (23.6%), and 103 (7.8%) patients included in stage 1–5 chronic kidney disease groups, respectively. The mean eGFR value was 49.3 ± 29.8 mL/min/1.73 m^2^, and the median adiponectin level was 10.7 (6.2–17.8) μg/mL. Metabolic syndrome was present in 585 (44.3%) patients. Patients were divided into 2 groups according to the presence of metabolic syndrome. UACR [208 (45–533) vs. 398 (113–1557) mg/g Cr] and hs-CRP [0.05 (0.02–0.18) vs. 0.12 (0.04–0.34) mg/dL] were higher, while eGFR (53.7 ± 30.9 vs. 43.8 ± 27.3 mL/min/1.73 m^2^) and adiponectin level [12.5 (7.1–19.6) vs. 8.6 (5.3–14.9) μg/mL] were significantly lower in the metabolic syndrome group compared to the non-metabolic syndrome group.

**Table 1:** Baseline characteristics of subjects

Variables	Total *n* = 1322	Without MS *n* = 737 (55.7%)	With MS *n* = 585 (44.3%)	*P*
Age (years)	53.5 ± 12.4	51.5 ± 12.7	56.1 ± 11.5	<0.001
Sex, men (%)	803 (60.7)	430 (58.3)	373 (63.8)	0.045
Smoking (%)	645 (48.8)	335 (45.5)	310 (53.0)	0.006
BMI (kg/m^2^)	24.4 ± 3.4	23.0 ± 2.9	26.1 ± 3.2	<0.001
Waist (cm)	87.1 ± 9.7	82.8 ± 8.6	92.6 ± 8.3	<0.001
SBP (mmHg)	128.0 ± 16.3	123.1 ± 14.1	134.1 ± 17.0	<0.001
DBP (mmHg)	76.8 ± 11.3	75.2 ± 10.7	78.8 ± 11.6	<0.001
CKD stage (%)				<0.001
Stage 1	153 (11.6)	107 (14.5)	46 (7.9)	
Stage 2	243 (18.4)	151 (20.5)	92 (15.7)	
Stage 3	511 (38.7)	285 (38.7)	226 (38.6)	
Stage 4	312 (23.6)	149 (20.2)	163 (27.9)	
Stage 5	103 (7.8)	45 (6.1)	58 (9.9)	
Cause of CKD (%)				<0.001
Glomerulonephritis	420 (31.8)	278 (37.7)	142 (24.3)	
Diabetes mellitus	295 (22.3)	87 (11.8)	208 (35.6)	
Hypertension	273 (20.7)	132 (17.9)	141 (24.1)	
PKD	239 (18.1)	181 (24.6)	58 (9.9)	
Laboratory findings				
Hemoglobin (g/dL)	12.8 ± 2.0	12.9 ± 1.9	12.7 ± 2.1	0.303
Albumin (g/dL)	4.19 ± 0.40	4.22 ± 0.37	4.15 ± 0.43	0.002
Glucose (mg/dL)	107.6 ± 36.0	98.7 ± 23.2	118.7 ± 45.0	<0.001
Uric acid (mg/dL)	7.12 ± 1.99	6.85 ± 1.94	7.47 ± 1.99	<0.001
Calcium (mg/dL)	8.96 ± 0.45	8.95 ± 0.43	8.96 ± 0.47	0.766
Phosphate (mg/dL)	3.70 ± 0.66	3.65 ± 0.61	3.78 ± 0.71	<0.001
Cholesterol (mg/dL)	173.8 ± 37.5	172.6 ± 35.4	175.4 ± 39.9	0.191
TG (mg/dL)	154.3 ± 96.2	113.8 ± 57.8	205.3 ± 109.7	<0.001
HDL-C (mg/dL)	49.6 ± 15.8	55.2 ± 15.4	42.7 ± 13.5	<0.001
LDL-C (mg/dL)	96.6 ± 30.7	96.5 ± 29.4	96.8 ± 32.2	0.889
hs-CRP (mg/dL)	0.07 (0.02–0.25)	0.05 (0.02–0.18)	0.12 (0.04–0.34)	<0.001
UACR (mg/g Cr)	273 (65–793)	208 (45–533)	398 (113–1557)	<0.001
eGFR (mL/min/1.73 m^2^)	49.3 ± 29.8	53.7 ± 30.9	43.8 ± 27.3	<0.001
Adiponectin (μg/mL)	10.7 (6.2–17.8)	12.5 (7.1–19.6)	8.6 (5.3–14.9)	<0.001
Male	9.1 (5.2–15.4)	10.5 (5.9–17.1)	7.7 (4.7–13.4)	<0.001
Female	13.1 (8.1–21.1)	14.4 (9.6–22.5)	10.8 (7.0–17.9)	<0.001
DM	10.5 (5.8–19.1)	13.4 (7.6–23.2)	8.0 (5.2–12.4)	0.001
Non-DM	10.8 (6.3–17.5)	12.4 (7.0–19.0)	9.7 (5.4–17.3)	<0.001
Co-morbidity, *n* (%)				
Hypertension	1215 (91.9)	651 (92.1)	564 (97.2)	<0.001
DM	425 (32.1)	125 (17.0)	300 (51.3)	<0.001
CVD	114 (8.6)	53 (7.2)	61 (10.4)	0.037
CAD	99 (7.5)	35 (4.7)	64 (10.9)	<0.001
PAD	24 (1.8)	9 (1.2)	15 (2.6)	0.069
CHF	11 (0.8)	4 (0.5)	7 (1.2)	0.194
Arrhythmia	29 (2.2)	16 (2.2)	13 (2.2)	0.950
Medication, *n* (%)				
ACEi	166 (12.6)	93 (12.6)	73 (12.5)	0.939
ARB	1046 (79.1)	562 (76.3)	484 (82.7)	0.004
Diuretics	432 (32.7)	171 (23.2)	261 (44.6)	<0.001
Beta blocker	368 (27.8)	142 (19.3)	226 (38.6)	<0.001
Statin	649 (49.1)	321 (43.6)	328 (56.1)	<0.001
Ezetimibe	95 (7.2)	42 (5.7)	53 (9.1)	0.019
Fibrate	31 (2.3)	7 (0.9)	24 (4.1)	<0.001

*MS* metabolic syndrome, *BMI* body mass index, *SBP* systolic blood pressure, *DBP* diastolic blood pressure, *CKD* chronic kidney disease, *PKD*, polycystic kidney disease, *TG* triglyceride, *HDL*-*C* high-density lipoprotein cholesterol, *LDL*-*C* low-density lipoprotein cholesterol, *hs*-*CRP* high sensitivity C-reactive protein, *UACR* urine albumin-to-creatinine ratio, *eGFR* estimated glomerular filtration rate, *DM* diabetes mellitus, *CVD* cerebrovascular disease, *CAD* coronary artery disease, *PAD* peripheral artery disease, *CHF* congestive heart failure, *ACEi* angiotensin converting enzyme inhibitor, *ARB* angiotensin II receptor blocker

**Fig. 1:**
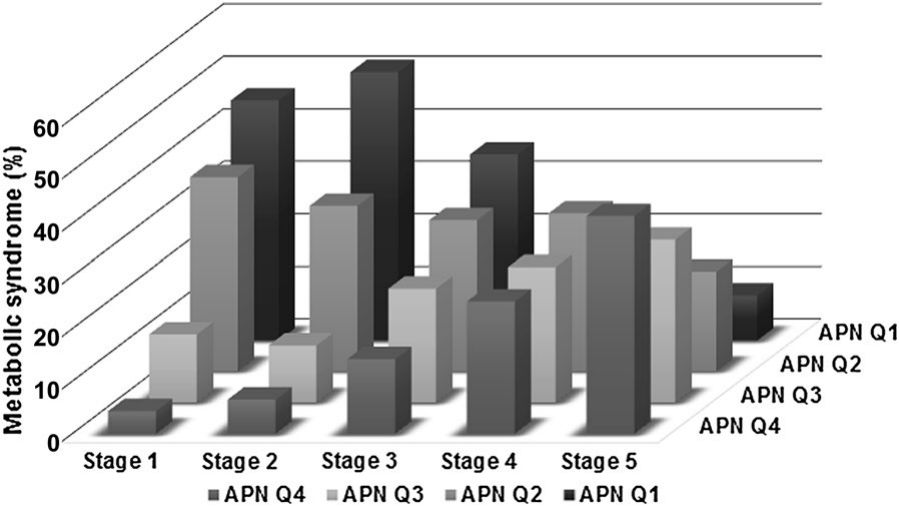
Crude prevalence rates of MS in the 20 groups, based on the combination of CKD stages and quartilized APN levels. Cross-categorization was performed based on the combination of CKD stages and quartilized APN category. Crude prevalence rates of MS were higher according to increased APN levels in CKD stage 1 [Q1 vs. Q4, 21 (45.7%) vs. 2 (4.3%)], stage 2 [47 (51.1%) vs. 6 (6.5%)], and stage 3 [80 (35.4%) vs. 32 (14.2%)] groups, but these correlations were blunted in stage 4. Furthermore, in stage 5 patients, group with the highest APN level had the most number of subjects diagnosed with metabolic syndrome [5 (8.6%) vs. 24 (41.4%)]. *MS* metabolic syndrome, *CKD* chronic kidney disease, *APN* adiponectin

Crude prevalence rates of metabolic syndrome, based on the combination of chronic kidney disease stages and quartilized adiponectin levels, are shown in Fig. 1. Cross-categorization was performed based on the combination of chronic kidney disease stages and quartilized adiponectin categories. Crude prevalence rates of metabolic syndrome were higher according to increased adiponectin levels in chronic kidney disease stage 1 [Q1 vs. Q4, 21 (45.7%) vs. 2 (4.3%)], 2 [47 (51.1%) vs. 6 (6.5%)], and stage 3 [80 (35.4%) vs. 32 (14.2%)] groups, but these correlations were blunted in stage 4 group. Furthermore, in stage 5 group, highest-level adiponectin group had the most number of subjects diagnosed with metabolic syndrome [5 (8.6%) vs. 24 (41.4%)].

### Relationship between clinical and biochemical variables and adiponectin level

Univariate linear regression analysis was performed to determine the relationship between clinical and biochemical variables and the adiponectin level (Table 2). In the analysis, age (β = 0.005), phosphate (β = 0.284), total cholesterol (per 10 mg/dL, β = 0.015), HDL-C (per 10 mg/dL, β = 0.116), UACR (per 1 log, β = 0.088), and diuretics (β = 0.141) were significantly positively related to adiponectin level, while sex (male, β = −0.366), smoking (β = −0.224), body mass index (β = −0.053), waist circumference (β = −0.020), hemoglobin (β = −0.159), serum albumin (β = −0.572), glucose (per 10 mg/dL, β = −0.012), calcium (β = −0.127), triglyceride (per 1 log, β = −0.332), hs-CRP (per 1 log, β = −0.050), eGFR (per 10 mL/min/1.73 m^2^, β = −0.066), and fibrate (β = −0.509) were negatively related with statistical significance.

**Table 2:** Linear regression analysis for the association of adiponectin with clinical and biochemical variables

Variables	Univariate		Multiple	
	β (95% CI)	*P*	β (95% CI)	*P*
Age (per 1 year)	0.005 (0.002 to 0.008)	0.004	0.002 (−0.002 to 0.005)	0.346
Sex (male)	−0.366 (−0.450 to −0.283)	<0.001	−0.067 (−0.196 to 0.061)	0.305
Smoking	−0.224 (−0.307 to −0.141)	<0.001	0.002 (−0.111 to 0.115)	0.975
Body mass index (per 1 kg/m^2^)	−0.053 (−0.065 to −0.041)	<0.001		
Waist circumference (per 1 cm)	−0.020 (−0.024 to −0.016)	<0.001	−0.008 (−0.013 to −0.003)	0.002
Systolic blood pressure (per 10 mmHg)	0.007 (−0.019 to 0.033)	0.584		
Diastolic blood pressure (per 10 mmHg)	−0.005 (−0.042 to 0.033)	0.811		
Laboratory findings				
Hemoglobin (per 1 g/dL)	−0.159 (−0.178 to −0.139)	<0.001	−0.069 (−0.099 to −0.039)	<0.001
Albumin (per 1 g/dL)	−0.572 (−0.672 to −0.473)	<0.001	−0.326 (−0.451 to −0.202)	<0.001
Glucose (per 10 mg/dL)	−0.012 (−0.024 to −0.001)	0.041	−0.009 (−0.021 to 0.003)	0.130
Uric acid (per 1 mg/dL)	0.014 (−0.007 to 0.036)	0.179		
Calcium (per 1 mg/dL)	−0.127 (−0.220 to −0.034)	0.008		
Phosphate (per 1 mg/dL)	0.284 (0.222 to 0.345)	<0.001		
Cholesterol (per 10 mg/dL)	0.015 (0.004 to 0.026)	0.008		
Triglyceride (per 1 log)^a^	−0.332 (−0.408 to −0.256)	<0.001	−0.192 (−0.283 to −0.101)	<0.001
HDL-C (per 10 mg/dL)	0.116 (0.090 to 0.142)	<0.001	0.097 (0.066 to 0.128)	<0.001
LDL-C (per 10 mg/dL)	0.009 (−0.005 to 0.023)	0.201		
hs-CRP (per 1 log)^a^	−0.050 (−0.076 to −0.024)	<0.001	−0.053 (−0.080 to −0.026)	<0.001
UACR (per 1 log)^a^	0.088 (0.062 to 0.113)	<0.001	0.028 (−0.001 to 0.056)	0.055
eGFR (per 10 mL/min/1.73 m^2^)	−0.066 (−0.080 to −0.052)	<0.001	−0.059 (−0.078 to −0.041)	<0.001
Co-morbidities				
Diabetes mellitus	0.018 (−0.072 to 0.108)	0.693		
Hypertension	−0.021 (−0.175 to 0.132)	0.786		
Coronary artery disease	0.071 (−0.088 to 0.230)	0.379		
Peripheral artery disease	0.116 (−0.198 to 0.429)	0.470		
Cerebrovascular disease	0.010 (−0.139 to 0.159)	0.897		
Congestive heart failure	−0.363 (−0.823 to 0.098)	0.122		
Arrhythmia	−0.156 (−0.441 to 0.130)	0.285		
Medications				
ACEi	0.045 (−0.082 to 0.171)	0.490		
ARB	−0.089 (−0.203 to 0.024)	0.123		
Diuretics	0.141 (0.051 to 0.231)	0.002	0.042 (−0.052 to 0.137)	0.377
Beta blocker	−0.059 (−0.153 to 0.035)	0.221		
Statin	−0.051 (−0.135 to 0.033)	0.236		
Ezetimibe	−0.081 (−0.243 to 0.081)	0.328		
Fibrate	−0.509 (−0.785 to −0.233)	<0.001	−0.358 (−0.627 to −0.089)	0.009

^a^Log transformed, adiponectin was also transformed by log

*HDL*-*C* high-density lipoprotein cholesterol, *LDL*-*C* low-density lipoprotein cholesterol, *hs*-*CRP* high sensitivity C-reactive protein, *UACR* urinary albumin-to-creatinine ratio, *eGFR* estimated glomerular filtration rate, *ACEi* angiotensin converting enzyme inhibitor, *ARB* angiotensin II receptor blocker

To further clarify the independent association of adiponectin level with clinical and biochemical variables, multiple linear regression analysis was performed with the variables that had significant differences in the comparison of baseline characteristics according to the presence of metabolic syndrome and univariate linear regression analysis. In the analysis, waist circumference (β = −0.020), hemoglobin (β = −0.069), serum albumin (β = −0.326), triglyceride (per 1 log, β = −0.192), HDL-C (per 10 mg/dL, β = 0.097), hs-CRP (per 1 log, β = −0.053), eGFR (per 10 mL/min/1.73 m^2^, β = −0.056), and fibrate use (β = −0.358) were independently related to adiponectin level.

### Adiponectin level is independently associated with the presence of metabolic syndrome

The relationship between the presence of metabolic syndrome and adiponectin level was evaluated by multiple logistic regression analysis with adjustments for age, sex, smoking status, body mass index, hemoglobin, serum albumin, uric acid, phosphate, hs-CRP, UACR, and eGFR. In addition, the past history of coronary artery disease and cerebrovascular disease, and current medication with ARB, diuretics, statins, ezetimibe, and fibrates were also adjusted (Table 3). In the fully adjusted model, decreased adiponectin levels were still independently associated with the risk of metabolic syndrome in chronic kidney disease patients [adiponectin, 1 μg/mL increase, odds ratio (OR), 95% confidence interval (CI) = 0.956 (0.937–0.976), *P* < 0.001]. Furthermore, the independent association was still significant in diabetic [OR (95% CI) = 0.942 (0.911–0.974), *P* < 0.001] or non-diabetic [OR (95% CI) = 0.967 (0.939–0.997), *P* = 0.031] nephropathy after adjustment for multiple confounding variables. For a more specific explanation of non-diabetic groups, hypertensive and glomerulonephritis groups still showed independent association between high adiponectin levels and presence of metabolic syndrome, while polycystic kidney disease group did not show any significance in the full adjustment model (Table 4).

**Table 3:** Adiponectin as a risk factor of metabolic syndrome

	Odds ratio (95% confidence interval)	
	Total	*P*
Crude	0.966 (0.954–0.978)	<0.001
Model 1	0.983 (0.969–0.997)	0.015
Model 2	0.961 (0.946–0.977)	<0.001
Model 3	0.959 (0.940–0.979)	<0.001
Model 4	0.956 (0.937–0.976)	<0.001

Adiponectin 1 μg/mL increase, subgroup analysis was performed according to the cause of chronic kidney disease

Model 1: adjusted for age, sex, smoking status, and body mass index

Model 2: adjusted for model 1 + hemoglobin, serum albumin, uric acid, and phosphate

Model 3: adjusted for model 2 + hs-CRP, urinary albumin-to-creatinine ratio, and eGFR

Model 4: adjusted for model 3 + coronary artery disease, cerebrovascular disease, ARB, diuretics, statin, ezetimibe, and fibrate

*DM* diabetes mellitus, *hs*-*CRP* high sensitivity C-reactive protein, *eGFR* estimated glomerular filtration rate, *ARB* angiotensin II receptor blocker

**Table 4:** Subgroup analyses of relationship between adiponectin levels and presence of metabolic syndrome

	Odds ratio (95% confidence interval)				
	DM	Non-DM			
		Total	HTN	GN	PKD
Crude	0.964 (0.943–0.984)	0.951 (0.935–0.967)	0.941 (0.912–0.971)	0.955 (0.931–0.979)	0.944 (0.904–0.987)
Model 1	0.973 (0.949–0.997)	0.969 (0.951–0.988)	0.949 (0.916–0.984)	0.980 (0.952–1.009)	0.954 (0.907–1.004)
Model 2	0.952 (0.923–0.981)	0.957 (0.935–0.980)	0.944 (0.905–0.985)	0.949 (0.913–0.987)	0.962 (0.911–1.016)
Model 3	0.946 (0.913–0.980)	0.957 (0.932–0.983)	0.950 (0.901–1.003)	0.942 (0.900–0.986)	0.950 (0.899–1.004)
Model 4	0.933 (0.898–0.970)	0.959 (0.932–0.986)	0.939 (0.885–0.996)	0.942 (0.896–0.990)	0.969 (0.913–1.028)

Adiponectin 1 μg/mL increase, subgroup analysis was performed according to the cause of chronic kidney disease

Model 1: adjusted for age, gender, smoking status, and body mass index

Model 2: adjusted for model 1 + hemoglobin, serum albumin, uric acid, phosphate, and parathyroid hormone

Model 3: adjusted for model 2 + hs-CRP, urine albumin-to-creatinine ratio, and eGFR

Model 4: adjusted for model 3 + coronary artery disease, cerebrovascular disease, ARB, diuretics, statin, ezetimibe, and fibrate

*DM* diabetic mellitus, *HTN* hypertension, *GN* glomerulonephritis, *PKD* polycystic kidney disease, *hs*-*CRP* high sensitivity C-reactive protein, *eGFR* estimated glomerular filtration rate, *ARB* angiotensin II receptor blocker

**Fig. 2:**
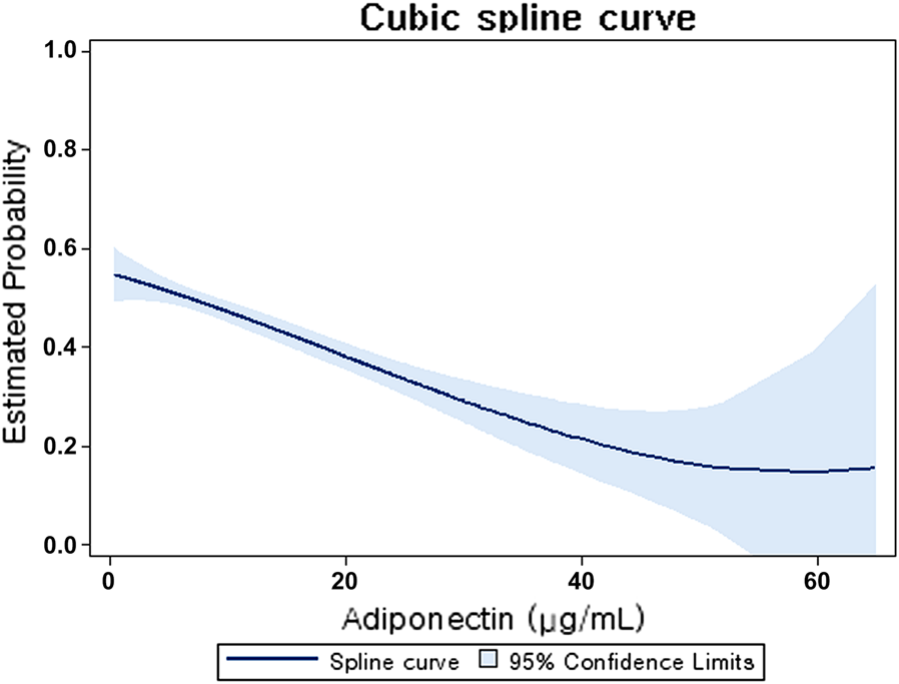
Cubic spline regression models of the estimated probability (with 95% confidence interval) of metabolic syndrome according to the adiponectin level. Multiple model adjusted for age, sex, smoking status, body mass index, hemoglobin, serum albumin, uric acid, phosphate, high sensitivity C-reactive protein, urinary albumin-to-creatinine ratio, estimated glomerular filtration rate, coronary artery disease, cerebrovascular disease, angiotensin II receptor blocker, diuretics, statin, ezetimibe, and fibrate

**Fig. 3:**
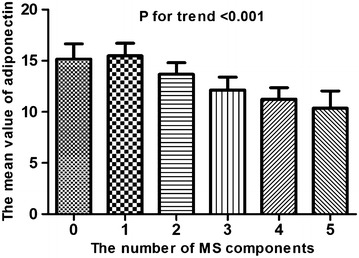
The mean value of adiponectin level according to the number of metabolic syndrome components. Each *bar* represents the mean and 95% confidence interval of adiponectin level. Adiponectin level are increased in parallel with the number of metabolic syndrome components (*P* for trend <0.001). *MS* metabolic syndrome

The results of the cubic splines graph illustrating the association between adiponectin levels and the risk of metabolic syndrome is shown in Fig. 2. A trend towards decreased association of metabolic syndrome was observed in chronic kidney disease patients with higher adiponectin levels. In addition, when the subjects were divided into 6 groups according to the number of metabolic syndrome components, the adiponectin level decreased as the number of components increased (*P* for the trend <0.001, Fig. 3).

## Discussion

In the general population, adiponectin may decrease the risk for metabolic disturbance-related diseases such as obesity, type 2 diabetes, and metabolic syndrome [16]. However, there has been an apparent paradox regarding its diverse correlations contributing to unfavorable clinical implications, including cardiac dysfunction, pulmonary disease, and chronic kidney disease. Although several existing theories suggest such paradox, the exact mechanism of hyperadiponectinemia in these disease entities remains incomprehensible. In particular, high serum adiponectin levels predict mortality and progression to end-stage renal disease in patients with kidney disease [17]. Given the opposite results between hyper- and hypoadiponectinemia on renal progression, cardiovascular outcomes, or metabolic disturbance-related disease, we have clarified the scope between circulating adiponectin levels and metabolic syndrome using a well-established chronic kidney disease cohort database. As a result, we clearly showed that hypoadiponectinemia was independently associated with the presence of metabolic syndrome, even after adjusting for multiple confounding factors including traditional risk factors, co-morbidities, and medication use.

In the present study, adiponectin level was negatively associated with eGFR, which is fairly consistent with previous investigations. Since the major clearance process of adiponectin occurs in the liver, explaining the exact mechanism of high adiponectin levels in low eGFR patients is not simple, and the causal relationship is unclear. In patients with chronic kidney disease, chronic inflammation, oxidative stress, and sympathetic overactivity are common clinical features, and these features might inhibit adiponectin expression [18]. However, the kidney has been regarded as a key organ for biodegradation of various proteins and cytokines; thus, decreased kidney function could promote the accumulation of adiponectin in the systemic blood. Although a counter-regulatory hyperadiponectinemia in acute pathologic conditions such as full-blown nephrotic syndrome has been reported [19], the evidence showing decreased adiponectin levels after kidney transplantation regardless of serum creatinine level or insulin resistance also strengthens the former postulation [20]. In addition, the ‘chronic’ damaged kidney function and consequent uremic milieu also results in hyperadiponectinemia through the inhibition of adiponectin gene expression and the activation of the renin-angiotensin system [21]. Meanwhile, relatively lower adiponectin levels compared to previous studies that investigated the impact of adiponectin levels in patients with chronic kidney disease might be due to the difference of mean eGFR in the present study population [22].

Our study showed that UACR was positively associated with adiponectin level. Even though the adipocyte is the chief secretory organ of adiponectin, the kidney is a major target organ through the adiponectin receptors (Adipo R) 1 and 2 in the intra-renal arterioles, endothelium, podocytes, mesangial cells of the glomerulus, and proximal tubular cells [23]. In experimental studies, adiponectin knockout mice showed exacerbation of albuminuria and renal fibrosis, and the restoration of adiponectin showed normalization of albuminuria, improvement of podocyte foot process effacement, and reduced urinary and glomerular markers of oxidant stress in adiponectin knockout mice [24]. In addition, the renal expression of Adipo R1 and R2 in adenine-induced chronic kidney disease rats is significantly increased compared to controls, and positively related to the adiponectin level in serum or urine [25]. All things taken together including previous mentioned evidence, the adiponectin level is substantially increased in full-blown nephrotic syndrome compared to controls [19], and it can be postulated that adiponectin is increased for counter-regulatory or kidney-protective purposes in damaged kidneys, similar to albuminuria.

From the clinical aspect, a landmark study showed that high adiponectin levels are associated with a lower risk of myocardial infarction in men [26], and this was followed by several remarkable studies that showed that higher adiponectin levels are associated with favorable clinical outcomes [27]. The favorable adiponectin effect is independently associated with not only eGFR and albuminuria, but also all-cause mortality and cardiovascular events in chronic kidney disease [11]. However, with established chronic kidney disease, the inverse relationship is not generally implicated and the findings are more complicated. The paradoxical increase of adiponectin level in those with the highest mortality may have been secondary to weight loss, which is a known stimulator of adiponectin as well as an independent risk factor in end-stage renal or heart disease patients [28]. The expression of adiponectin receptor mRNA on peripheral blood mononuclear cells in end-stage renal disease patients on hemodialysis [29] is increased and significantly related to subcutaneous and visceral fat. These findings imply that the inverse relationship between adiponectin level and renal function could result from the resistance effect of adiponectin in patients with chronic uremia, thus the regulation of expression, secretion, or excretion of adiponectin is destroyed [30].

Nevertheless, the present analysis indicates that adiponectin level was independently associated with the risk of metabolic syndrome in chronic kidney disease patients after adjustment for confounding factors including eGFR and UACR. Adiponectin is secreted from visceral fat. Therefore, a strong relationship between adiponectin level and metabolic components including triglycerides, HDL-C, and waist measurements might overwhelm the association with renal function. In particular, waist enlargement is the second-most common metabolic syndrome component, followed by glucose. In this analysis, the relationship between adiponectin level and the risk of metabolic syndrome would mostly be influenced by the prevalence of larger-waist subjects. In obese subjects, adiponectin levels were lower despite the fact that adipose tissue is its source [31]. In addition, adiponectin level was demonstrated to be lower in body weight reduction subjects [32]. Waist circumference is not only a simple indicator of obesity but is also related to non-traditional risk factors such as inflammation [33]. The strong relationship between metabolic derangements such as waist circumference or hypertriglyceridemia and adiponectin level suggests that these metabolic factors are overwhelming the association between adiponectin level and low eGFR or albuminuria in patients with chronic kidney disease.

In the end, we found several results. First, serum albumin levels were significantly and independently associated with adiponectin levels in our analysis. In fact, this clear finding has already been reported in several previous investigations [11, 34], and the mechanistic linkage have postulated through protein-energy wasting syndrome (PEWS), which is the chief contributor to adverse clinical outcomes in uremic patients [17]. Given that serum albumin levels have been regarded as major markers of either malnutrition or PEWS, the inverse relationship between serum albumin and circulating adiponectin concentrations might be in line with our findings. Second, low hemoglobin levels were independently associated with high adiponectin levels, and these findings have also been reported in several previous investigations [35, 36]. It remains uncertain why low hemoglobin concentrations are independently associated with high circulating adiponectin levels. One plausible explanation is the increased expression of hypoxia inducible factor (HIF), which results from anemia-induced tissue hypoxia. A recent experimental study clearly indicated that HIF-1 activation with a variety of stimuli significantly increases adiponectin expression [37]. Another suggestion is that circulating adiponectin would be chiefly secreted from bone marrow fat, with an inverse correlation between fat mass and circulating adiponectin levels [38]. Considering that marrow adipocyte is a negative regulator of hematopoiesis [39], we would assume an independent relationship between circulating adiponectin and hemoglobin levels.

Several limitations of our study must be acknowledged. First, the study was performed with an analysis of cross-sectional data, the causative role of adiponectin on development of metabolic syndrome is still inconclusive. Thus, given the differences in overall outcome between previous studies, further research with a prospective design in this area should be prioritized. Second, in the course of defining metabolic syndrome, the waist circumference was evaluated using Asian criteria. Although there is controversy about defining the waist criteria in metabolic syndrome diagnosis, the Korean Diabetes Association presented Korean waist criteria, which is the same as the Asian criteria in the harmonizing definition. Third, the genetic polymorphism of adiponectin levels was also reported [40], thus the fact that a single ethnicity Korean cohort was used should be considered during interpretation.

## Conclusion

In conclusion, low adiponectin levels were independently associated with the presence of metabolic syndrome in chronic kidney disease patients. The relationship showed linearity throughout the categorical adiponectin levels. A strategy to modulate serum adiponectin might be helpful to improve metabolic derangements in patients with chronic kidney disease.
